# 

**Published:** 1895-05-25

**Authors:** 


					Jj^jai/a'P773H7^M"MTnppliea tinder Royal Warrant to Her Majesty the Qneen. May 25th, 1895.
y*
/ Tr"*"" -VWJM* ?? Ml? * MP? W WW MV* *Mil?jVa UJ W**v ^ UWWUl *?*?* W**J AUWU.
? " THE KING OF NATURAL TABLE WATERS. "T "1 ? *!
tloi^onm^ tH| Jo^anim
IRW/CH HOSf.LTAl
No. 452. vol xvixi. WITH EXTRA NURSING SUPPLEMENT. price twopence.
aTUrday May 25 lg [Registered at the General Post Office as a Newspaper for MontwyTarls
~  ' tranMniiston In the United Kingdom and AbroadJ

				

